# An Exceedingly Rare Presentation of Severe Folate Deficiency-Induced Non-Immune Hemolytic Anemia

**DOI:** 10.7759/cureus.8570

**Published:** 2020-06-11

**Authors:** Qian Zhang, Khine S Shan, Babatunde A Ogunnaike, Atsoufui Amewuame-Kpehor, Travis Nace

**Affiliations:** 1 Internal Medicine, Abington Hospital-Jefferson Health, Abington, USA; 2 Internal Medicine, University of Maryland Medical Center, Baltimore, USA; 3 Library Science, Abington Hospital-Jefferson Health, Abington, USA

**Keywords:** folate deficiency, anemia, non-immune hemolytic anemia, intramedullary hemolysis, megaloblastic anemia

## Abstract

Macrocytic anemia is usually associated with vitamin B12 or folate deficiency. However, folate deficiency was rarely reported as a cause of hemolytic anemia. We present a case of a young man with a history of alcohol abuse who initially presented with an acute on chronic abdominal pain and was found to have jaundice and scleral icterus. His liver enzymes were unremarkable, and his abdominal imaging did not reveal any acute pathology. However, he was found to have a severe non-immune hemolytic anemia secondary to folate deficiency.

## Introduction

Anemia is a global common health problem that results in non-specific symptoms, such as fatigue, dizziness, or dyspnea, due to a decreased amount of red blood cells (RBC) to provide oxygenation to the body tissues [1]. Anemia is further categorized into microcytic, normocytic, and macrocytic based on the mean corpuscular volume (MCV). Macrocytic anemias occur when the MCV is greater than 100 fL. Macrocytic anemia is classified into megaloblastic or non-megaloblastic anemia based on findings on the peripheral blood smear. Megaloblasts and hypersegmented neutrophils are common findings of macrocytic anemia, while the presence of Howell-Jolly bodies, anisocytosis, and poikilocytosis are also seen [2]. Vitamin B12 or folate deficiency is the most common cause of megaloblastic anemia as there is a deficiency or an impaired utilization of the vitamins that affect the DNA synthesis, which results in a premature release of RBCs from the bone marrow. Non-megaloblastic anemia is correlated with liver dysfunction, alcoholism, myelodysplastic syndrome (MDS), and hypothyroidism [3].

## Case presentation

Our patient is a 39-year-old gentleman who initially presented to the hospital with a chief complaint of acute on chronic abdominal pain for the past four days. His past medical history was significant for gastritis and peptic ulcer disease confirmed on endoscopy six years ago. He worked as a truck driver and rarely seek medical attention prior to his presentation to his primary care physician eight months ago with laboratory findings of non-specific anemia that was not followed upon. He denied any outpatient medication use or prior blood transfusions. He denied a history of smoking and illicit drug use but admitted to heavy chronic alcohol use over the past 15 years. He decided to present to the hospital as he had an acute worsening of chronic generalized abdominal pain accompanied by nausea and vomiting without bloody vomitus.

**Figure 1:**
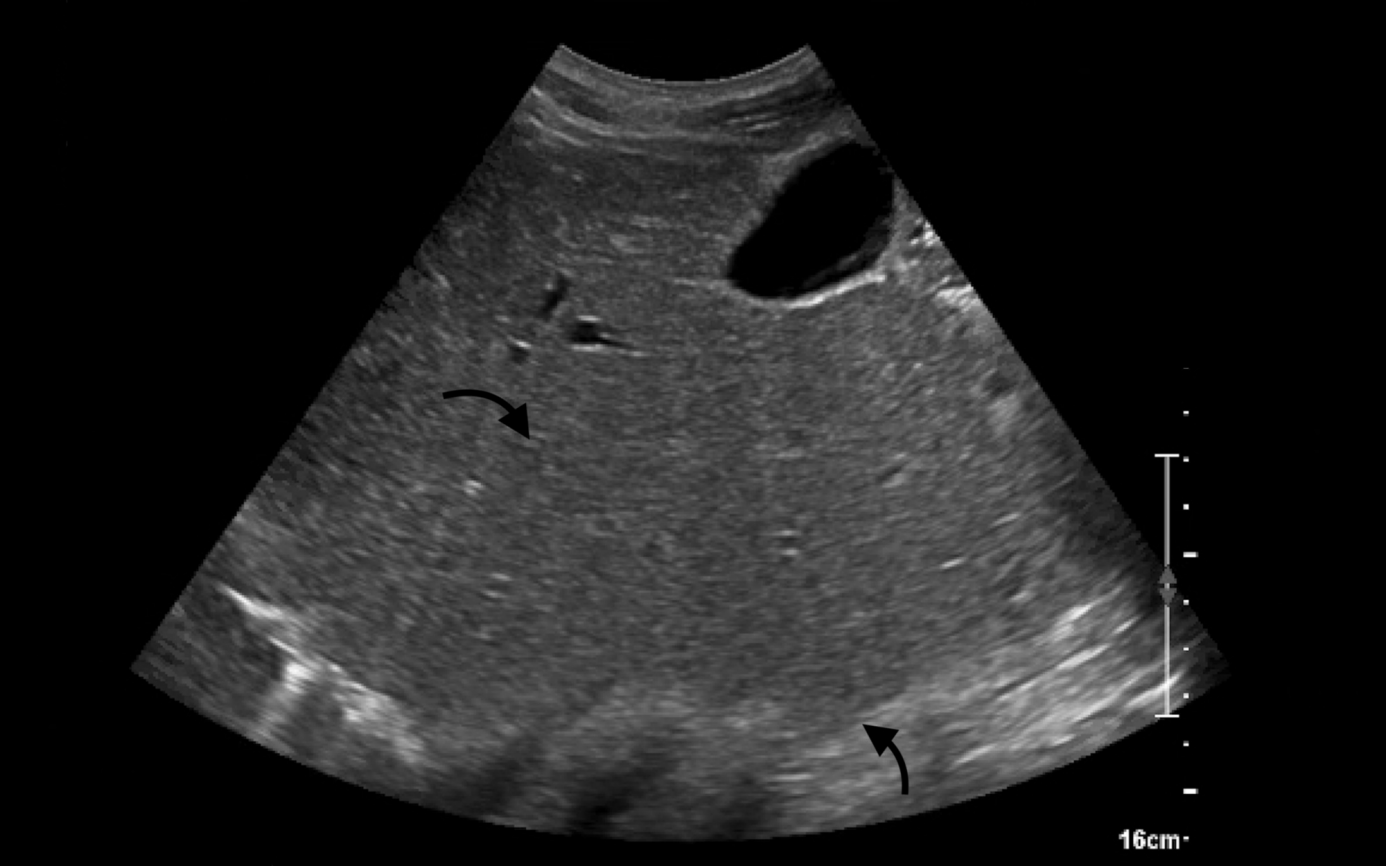
Ultrasound of the Liver Mild coarsened echotexture without evident nodularity, architectural distortion, masses, or intrahepatic biliary ductal dilation.

On presentation to the emergency department, his vital signs were as follows: temperature 98.1°F, blood pressure 114/74 mmHg, respiratory rate 18 breaths per minute, heart rate 80 beats per minute, and oxygen saturation 100% on room air. His review of systems was significant for recent unintentional weight loss and dark urine. Physical examination was unremarkable except for generalized jaundiced skin with scleral icterus that reportedly had been present over the past six months and minimal epigastric tenderness on abdominal palpation. Pertinent laboratory findings included aspartate transaminase (AST) 54 U/L, alkaline phosphatase 66 U/L, total bilirubin 9.0 mg/dL, direct bilirubin 0.3 mg/dL, lipase 24 U/L, hemoglobin 5.8 g/dL, MCV 124 K/UL, mean corpuscular hemoglobin (MCH) 47.1 pg, red blood cell distribution width (RDW) 31.7%, platelets 124 K/UL, international normalized ratio (INR) 1.7 (not on warfarin), and partial thromboplastin time (PTT) 29 seconds. Urinalysis was positive for elevated urobilinogen 2.0 mg/dL. Peripheral blood smear showed moderate macroovalocytes and occasional Howell-Jolly bodies, despite no splenectomy history or findings of functional asplenia. CT of the abdomen and pelvis with contrast was unremarkable for intra-abdominal pathologies or lymphoproliferative disorders. Ultrasound of the liver showed coarsened hepatic echotexture consistent with a history of alcohol-induced hepatic steatosis (Figure 1). He was transfused with two packs of RBCs due to the low hemoglobin, despite no active signs of bleeding. He remained hemodynamically stable overnight.

On day 2 of hospitalization, hemoglobin was 7.8 g/dL post-transfusion. Additional laboratory findings showed lactate dehydrogenase (LDH) 1,850 U/L, total protein 5.3 gm/dL, reticulocyte percentage of 47.8%, absolute reticulocyte 1,031 K/UL, haptoglobin <3 mg/dL, vitamin B12 >2,000 pg/mL, folate <2.5 ng/mL, glucose-6-phosphate-dehydrogenase (G6PD) 23.5 U/g Hgb, unremarkable smooth muscle antibody, and alpha-1-antitrypsin antibody. His abdominal pain and vomiting improved without signs of acute gastrointestinal blood loss. He had stable hemodynamics, along with a negative fecal occult test; thus, emergent endoscopy was deferred. The clinical impression was consistent with hemolytic anemia given elevated reticulocyte count and LDH, indirect hyperbilirubinemia, undetectable haptoglobin in the setting of no recent medication changes, and no signs of active infection. His folate deficiency was possibly related to malnutrition and chronic alcoholism. It was determined to treat the folate deficiency with intravenous folate two milligrams (mg) daily. Furthermore, the Coombs antibody test was ordered to further investigate the underlying cause of hemolytic anemia. Thrombocytopenia was thought to be related to folate deficiency. 

On day 3 of hospitalization, hemoglobin remained to be stable at 7.9 g/dL without signs of bleeding. The Coombs antibody test was negative, thus indicating non-immune hemolytic anemia. The hemoglobin electrophoresis interpretation showed normal adult type A hemoglobin pattern. Abdominal pain and vomiting had resolved completely. The patient was deemed to be stable to be discharged from the hospital with oral folic acid 5 mg daily and was advised to achieve alcohol abstinence as well as follow up with a hematologist in the outpatient setting for the continuation of care.

One week after hospital discharge, the patient’s hemoglobin remained to be stable at 9.3 g/dL without signs of bleeding. Other pertinent laboratory findings included MCV 108 K/UL, platelets 515 K/UL, total bilirubin 1.9 mg/dL, LDH 875 U/L, haptoglobin 37 mg/dL, folate 7.0 ng/mL, vitamin B12 293 pg/mL, and negative paroxysmal nocturnal hemoglobinuria (PNH) flow cytometry testing with fluorescein-labeled proaerolysin (FLAER). Given his recent presentation of profound macrocytosis and laboratory features suggestive of non-immune mediated hemolysis with clinical response to folate repletion, the clinical impression at the time was thought to be related to folate deficiency. He was recommended to continue oral folic acid 5 mg daily and schedule a follow-up appointment in one month.

The patient reported feeling much better overall after receiving folate replacement one month after the initial outpatient encounter. He also began to follow a healthy diet and denied further episodes of abdominal discomfort that subsequently led to a 10 pounds weight gain. He continued to take daily folic acid and had been abstinent from alcohol use. Physical examination revealed an improvement of jaundice compared to prior visits. Pertinent laboratory findings included hemoglobin 11.0 g/dL, MCV 93.6 K/UL, platelets 338 K/UL, total bilirubin 0.5 mg/dL, LDH 214 U/L, haptoglobin 114 mg/dL, and folate 18.9 ng/mL. He was recommended to continue oral folic acid 5 mg daily as his clinical status and laboratory findings improved drastically status post folate repletion along with alcohol use cessation.

## Discussion

Our patient was an otherwise healthy young gentleman without medical comorbidities who presented with acute on chronic abdominal pain that was later discovered to be most likely due to a gastroenterology source. However, his initial laboratory findings showed profound macrocytic anemia with peripheral blood smear findings of moderate macroovalocytes and occasional Howell-Jolly bodies. Vitamin B12 and folate levels were checked appropriately, which showed a folate level <2.5 ng/mL with normal vitamin B12 levels. Folic acid is found in green vegetables and animal products. It is absorbed in the upper jejunum of the gastrointestinal tract. A decreased level of folate is often correlated with a nutritional deficiency in the setting of poor diet or alcoholism. Other possible causes are gastrointestinal pathologies that affect the proper absorption of folate, increased body’s metabolic requirements (e.g. pregnancy, chronic hemolysis), or pharmacological alteration by certain medication’s mechanisms of action (e.g. methotrexate, trimethoprim, phenytoin) [3]. It was assumed that our patient’s folate deficiency anemia was due to the history of chronic alcoholism and possible malnutrition.

However, the more concerning laboratory finding of our patient was the initial hemoglobin of 5.8 g/dL that required blood transfusion along with other significant markers correlated with hemolysis. Hemolytic anemia occurs when RBCs are prematurely destroyed prior to their normal 120 days of life span intravascularly or extravascularly. There are various causes of hemolytic anemia, which include hemoglobinopathies, inherited protein defects, enzymopathies, immune-mediated, infections, direct traumas, or drug-induced. Initial hemolysis workup typically includes LDH, haptoglobin, unconjugated bilirubin, reticulocyte counts, and urinalysis [4]. Our patient had elevated LDH, unconjugated bilirubin, reticulocyte counts, urobilinogen, and decreased haptoglobin that correlated with the findings of hemolytic anemia. A peripheral blood smear is often obtained next in order to identify the specific etiology of hemolytic anemia in attempting to identify abnormal RBCs, such as spherocytes, schistocytes, and bite cells [4]. However, our patient only had peripheral blood smear findings of macroovalocytes and Howell-Jolly bodies that were significant for macrocytic anemia. Extensive hemolytic anemia workup was initiated despite an unconventional peripheral blood smear that included the direct Coombs test, glucose-6-phosphate-dehydrogenase (G6PD), hemoglobin electrophoresis, and flow cytometry testing with FLAER. The results were unremarkable. There were no candidate medications to implicate as a cause of hemolysis either.

Autoimmune hemolytic anemia (AIHA) is an uncommon disorder caused by autoantibodies directed against self RBCs that could be idiopathic (50%) or associated with lymphoproliferative syndromes (20%), autoimmune disorders (20%), infections, or tumors. AIHA is further classified into warm, cold, or mixed categories based on the thermal range of the autoantibody [5]. Diagnosis is achieved based on findings of the direct antiglobulin test (DAT) that demonstrates the presence of autoantibodies or complements on the surface of the RBCs [6]. Our patient’s anemia appeared to be non-immune mediated given a negative direct Coombs test. In addition, false-negative results may occur in less than 10% of DAT due to IgA autoantibodies (not detectable by most routine reagents), low-affinity IgG, or RBC-bound IgG below the threshold of the test [5]. However, our patient had drastic improvement of symptoms, physical examination findings, and laboratory data status post folate repletion a few months after the initial encounter with close outpatient follow-up appointments. He had significant evidence of intramedullary hemolysis from megaloblastosis was able to mount proper bone marrow responses given elevated reticulocyte counts.

Nevertheless, the patient’s folate deficiency could be due to brisk hemolysis with compensatory hemolysis. Folate deficiency is often seen when there is an increased body’s demand for folate in circumstances such as pregnancy, puberty, or hemolytic anemia [7]. However, there was a lack of evidence or clinical impression to suggest this as our patient had unremarkable workups, insignificant past medical or social history, and a prominent response to the folate replacement.

There is currently a lack of published reports that link severe folate deficiency with non-immune hemolytic anemia based on our literature review. Cheema et al. reported a case of an unusual presentation of vitamin B12 deficiency-induced hemolytic anemia [8]. The patient was a 59-year-old male with no significant past medical history who was found to have hemolytic anemia with vitamin B12 level of 52 pg/mL. One proposed mechanism of action is that the elevated homocysteine levels found in vitamin B12 deficiency could have pro-oxidant properties that cause endothelial damage with subsequent microangiopathy [9,10]. Both vitamin B12 and folate are responsible for the remethylation process that converts homocysteine to methionine. A lack of either of them will lead to ineffective hematopoiesis with subsequent accumulation of homocysteine [8]. One of the limitations of our case report is that the homocysteine level was not checked initially during hospitalization since other hemolytic anemia workups were pending and the patient responded to folate replacement during the outpatient follow-up appointments. Furthermore, the case report described by Cheema et al. had a normal homocysteine level prior to the initiation of vitamin B12 therapy [8].

## Conclusions

Folate deficiency could be a rare entity of hemolytic anemia despite there is currently a lack of research that details the underlying mechanisms of action. Intramedullary hemolysis leading to fragile RBCs could be possibly related to this phenomenon. Close attention should be paid to the possible connection of folate deficiency and hemolytic anemia by a provider as it could be critical in investigating the potential underlying cause of hemolysis. 
